# Antifungal Activity of Ribosome-Inactivating Proteins

**DOI:** 10.3390/toxins16040192

**Published:** 2024-04-15

**Authors:** Rosario Iglesias, Lucía Citores, Claudia C. Gay, José M. Ferreras

**Affiliations:** 1 Department of Biochemistry and Molecular Biology and Physiology, Faculty of Sciences, University of Valladolid, E-47011 Valladolid, Spain; riglesias@uva.es (R.I.); lucia.citores@uva.es (L.C.); 2 Laboratory of Protein Research, Institute of Basic and Applied Chemistry of Northeast Argentina (UNNE-CONICET), Faculty of Exact and Natural Sciences and Surveying, Av. Libertad 5470, Corrientes 3400, Argentina; claudiacgay@exa.unne.edu.ar

**Keywords:** adenine polynucleotide glycosylase, antifungal protein, fungus-resistant transgenic plants, plant pathogenic fungi, ribosome-inactivating protein (RIP), rRNA glycosylase (EC 3.2.2.22)

## Abstract

The control of crop diseases caused by fungi remains a major problem and there is a need to find effective fungicides that are environmentally friendly. Plants are an excellent source for this purpose because they have developed defense mechanisms to cope with fungal infections. Among the plant proteins that play a role in defense are ribosome-inactivating proteins (RIPs), enzymes obtained mainly from angiosperms that, in addition to inactivating ribosomes, have been studied as antiviral, fungicidal, and insecticidal proteins. In this review, we summarize and discuss the potential use of RIPs (and other proteins with similar activity) as antifungal agents, with special emphasis on RIP/fungus specificity, possible mechanisms of antifungal action, and the use of RIP genes to obtain fungus-resistant transgenic plants. It also highlights the fact that these proteins also have antiviral and insecticidal activity, which makes them very versatile tools for crop protection.

## 1. Introduction

Diseases caused by plant pathogens are continuously increasing, causing severe losses in agricultural production, as disease prevalence can reach 70–80% of the total plant population and yields can decrease in some cases up to 80–98% [1]. The main phytopathogens are viruses, bacteria, and fungi [1]. Fungi are responsible for 80% of plant diseases [2] and fungal epidemics have had significant social and economic repercussions throughout history and today [3]. To control these diseases, chemical-based fungicides are used, which are very effective but bring with them problems such as environmental contamination, development of resistance, and residual toxicity [2,4,5]. Therefore, the fight against fungal diseases remains a major challenge and there is a need to find effective fungicides that are environmentally friendly. In this context, the search for more effective and safer fungicides continues to be a field of intense research. Plants are one of the most widely used sources, as they have developed various protein-based defense mechanisms to cope with fungal infections. However, the control of crop diseases using this type of fungicides has drawbacks, such as the instability of many of these agents in the field [6] and their high cost of production [2]. A solution to these drawbacks may be the use of transgenic plants carrying genes that code for antifungal proteins. Antifungal proteins include chitinases, glucanases, thaumatin-like proteins, thionins, cyclophilin-like proteins, lectins, ribonucleases, deoxyribonucleases, peroxidases, protease inhibitors, and ribosome-inactivating proteins [7,8]. Ribosome-inactivating proteins (RIPs) may be an excellent choice, as they have been attributed a defense role in plants and have been shown to have great potential against viruses, fungi, and insects [9,10,11,12].

In this review, we summarize and discuss the potential use of ribosome-inactivating proteins (RIPs) for agricultural applications from a bioengineering and biotechnology perspective, with special emphasis on RIP/fungus specificity, possible mechanisms of antifungal action, and the use of RIP genes to obtain transgenic plants resistant to fungi.

## 2. Ribosome-Inactivating Proteins

Ribosome-inactivating proteins (RIPs) are a group of proteins that inactivate ribosomes, leading to irreversible inhibition of protein synthesis (with IC_50_ values, i.e., concentration inhibiting protein synthesis by 50%, for animal cell-free systems in the range of 0.015 to 3.5 nM) and, consequently, cell death [13,14,15,16,17]. RIPs have been classified according to their structure into type 1 RIPs, consisting of a polypeptide chain with enzymatic activity, and type 2 RIPs, made up of two polypeptide chains, an A chain with enzymatic activity and a B chain with lectin activity that can bind to cell surface receptors facilitating RIP entry [13]. In addition, a third class of RIPs, termed type 3 RIPs, has been recognized, which includes a few members, such as jasmonate-induced protein (JIP60) and maize b-32 protein, which are activated by proteolysis [13,14,16,17]. Some type 2 RIPs such as ricin and abrin are extremely toxic (with IC_50_ values for cell cultures between 0.3 and 8 pM), while others have low toxicity, because the binding of the B-chain to oligosaccharides on the cell surface is less efficient and because, once internalized, the RIP follows a different intracellular pathway than ricin [16]. The toxicity of type 1 RIPs is lower (with IC_50_ values for cell cultures between 2 and 34 µM) since they lack the lectin part and are therefore unable to bind to cells as type 2 RIPs do. The structure, activity, and mode of action of RIPs have been studied over the last decades, but their biological function has not been demonstrated, although there is a broad consensus that these proteins play an important role in the defense of plants against viruses, fungi, and insects [9,12,18].

Due to their diverse activities, RIPs, alone or as part of conjugates, are good candidates for developing selective antiviral and anticancer agents [12,19,20,21,22]. Conjugates consist of a targeting moiety, such as an antibody, lectin, or growth factor, linked to a toxic moiety. RIPs have been used as the toxic part in several conjugates that have been tested in experimental therapies against various malignancies. In agriculture, RIPs have been shown to increase resistance against viruses, fungi, and insects in transgenic plants [9,12,21].

RIPs are present in a large number of angiosperm plants, both monocotyledonous and dicotyledonous, although in some plant families it is more common to find RIPs than in others, thus there are families such as the Poaceae, Euphorbiaceae, Cucurbitaceae, Caryophyllaceae, Amaranthaceae, and Phytolacaceae, where several species have been found with RIPs, and other families where they have never been found [13,14,23]. Some bacteria possess toxins with rRNA *N*-glycosylase activity whose sequence is homologous to plant RIPs [24]. The best known are Shiga toxin and related proteins consisting of an A chain with *N*-glycosylase activity and a B subunit, which is a pentamer that binds to specific glycolipids of the plasma membrane facilitating their endocytosis [25].

Although RIPs were initially studied as inhibitors of mammalian ribosomes, they can also inactivate ribosomes from other animals [26] and fungi [27,28,29,30], and, in some cases, ribosomes from bacteria [9] and plants [31]. The ability to inactivate plant ribosomes is, as we will discuss later, of particular significance in the defense against pathogens. Table 1 identifies the RIPs that inactivate or do not inactivate ribosomes of various plant species.

The inhibitory activity of RIPs on plant ribosomes is very diverse. It appears that RIPs from Poaceae do not inhibit protein synthesis in plants, or, if they do (OsRIP1 in the germ system of wheat or tritin-L in wheat and tobacco), it is at very high concentrations. Neither type 2 RIPs from *Abrus precatorius* L. or *Viscum album* L., nor RIPs from the genus *Sambucus*, whether type 1 or 2, inhibit protein synthesis in plants. Type 2 RIPs from *Ricinus comunis* L. (ricin and RCA) inhibit protein synthesis in different plant germ systems but do so at very high concentrations. The case of cucurbits is inconclusive because only bryodin has been tested in the germ-derived system of *Cucumis sativus* L. However, type 1 RIPs from the Phytolaccaceae, Amaranthaceae, and Caryophyllaceae families inhibit protein synthesis in practically all systems in which they have been tested in the nM range, a concentration which, given the reported yield for purification of many RIPs, could easily be achieved in vivo. The reason for not inactivating their ribosomes is that RIPs of dicots, like those of monocots of the family Asparagaceae, have a leader peptide, are synthesized in the endoplasmic reticulum and exported to the apoplast, thus avoiding contact with ribosomes [32]. In this regard, it should be noted that the RIPs from *Phytolacca americana* L. and *Phytolacca dodecandra* L’Hér. have been tested on their ribosomes, which are sensitive to the toxins [33].

**Table 1:** Sensitivity of plant ribosomes to ribosome-inactivating proteins. The families and species of both the RIP source (rows) and the ribosome source (columns) are indicated.

RIP	IC_50_ * (nM)							References
	Cucurbitaceae	Brassicacea	Euphorbiaceae	Fabaceae	Phytolaccaceae	Poaceae	Solanaceae	
AMARANTHACEAE								
*Beta vulgaris* L.								
BE27				Yes ** (VS)				[30,34]
ASPARAGACEAE								
*Agave tequilana* F.A.C.Weber								
Mayahuelin						10.43 (TA)		[35]
*Asparagus officinalis* L.								
Asparin 1	1333 (CM)			No * (VS)				[36,37]
*Muscari armeniacum* H.J.Veitch								
Musarmins 1-2-3				No * (VS)		No * (TA)		[38]
CARYOPHYLLACEAE								
*Dianthus caryophyllus* L.								
Dianthin 30	No * (CM)							[37]
Dianthin 32							Yes ** (NT)	[39]
*Saponaria officinalis* L.								
Saporin-L1	26.7 (CM) 17.3 (CS)			0.99 (VS)		40 (TA)		[37,40]
Saporin-L2	31 (CS)			20.91 (VS)		23.7 (TA)		[40]
Saporin-R1	1105 (CS)			0.22 (VS)		582 (TA)		[40]
Saporin-R2	55.7 (CS)			0.97 (VS)		3.1 (TA)		[40]
Saporin-R3	959 (CS)			0.02 (VS)		176 (TA)		[40]
Saporin-S5	10 (CM) 0.03 (CS)			0.34–0.48 (VS)		772 (TA)		[31,37,40,41]
Saporin-S6	3606 (CS)			0.31 (VS)		32 (TA)		[40]
*Silene glaucifolia* Lag. (=*Petrocoptis glaucifolia* Boiss.)								
Petroglaucin 1	219 (CS)			49 (VS)		30 (TA)		[42]
Petroglaucin 2	27–29 (CS)			0.2–6 (VS) 127 (VL)		30–73 (TA)		[36,42,43]
*Silene laxipruinosa* Mayol and Rosselló (=*Petrocoptis grandiflora* Rothm.)								
Petrograndin	186 (CS)			5 (VS)		100(TA)		[42]
CUCURBITACEAE								
*Bryonia dioica* Sessé and Moc.								
Bryodin	No * (CS)							[41]
EUPHORBIACEAE								
*Ricinus communis* L.								
Ricin	1473 (CL)		1470 (RC)	923 (PS)	1700 (PA)	1313 (TA) 980 (HV)	Yes ** (NT)	[33,39,44]
RCA	No * (CM) 3767 (CL)		8167 (RC)	No * (VS) 7500 (PS)		19,333 (TA) 7567 (HV)		[44,45]
FABACEAE								
*Abrus precatorius* L.								
APA	No * (CM)			No * (VS)		No * (TA)		[45]
PHYTOLACCACEAE								
*Phytolacca americana* L								
PAP (PAP I)					1.1–2.9 (PA)	0.3 (TA)	Yes ** (NT)	[33,39]
PAP II					3.9 (PA)			[33]
PAP-S	54–500 (CS)			0.26–0.38 (VS)	6.7 (PA)	4.5 (TA)		[31,33,36,41,46]
*Phytolacca dioica* L.								
PD L4-S2, Dioicin 2				Yes ** (VS)				[30]
*Phytolacca dodecandra* L’Hér.								
Dodecandrin					0.8–3.1 (PD)	0.2 (TA)		[33]
POACEAE								
*Hordeum vulgare* L.								
Barley RIP 30							No ** (NT)	[39]
*Oryza sativa* L.								
OsRIP1						1500 (TA)		[47]
*Triticum aestivum* L.								
Tritin (Tritin-S)		No ** (AT)		No ** (LJ)		No ** (TA)	No ** (NT)	[39,48]
Tritin-L		Yes ** (AT)		Yes ** (LJ)		Yes ** (TA)	Yes ** (NT)	[48]
*Zea mays* L.								
pro-RIP, αβ RIP						No ** (ZM)		[49]
SANTALACEAE								
*Viscum album* L.								
VAA	No * (CM)			No * (VS)		No * (TA)		[45]
VIBURNACEAE								
*Sambucus ebulus* L.								
Ebulin f	No * (CS)			No * (VS)		No * (TA)		[50]
Ebulin r1–r2	No * (CM)			No * (VS)		No * (TA)		[50]
α-β-γ-Ebulitin	No * (CM)			No * (VS)		No * (TA)		[50]
*Sambucus nigra* L.								
Nigrin b	No * (CS)			No * (VS)		No * (TA)		[50]
Nigrin f	No * (CS)			No * (VS)		No * (TA)		[50]
basic Nigrin b						No * (TA)		[50]
Nigritin f1–f2	No * (CS)			No * (VS)		No * (TA)		[50]

The table lists both protein synthesis inhibition assays (IC_50_ is indicated, i.e., concentration inhibiting protein synthesis by 50%) * and *N*-glycosylase activity assays ** on ribosomes of the following species: *Arabidopsis thaliana* (L.) Heynh (AT); *Citrullus lanatus* (Thunb.) Matsum. and Nakai (CL); *Cucumis melo* L. (CM); *Cucumis sativus* L. (CS); *Hordeum vulgare* L. (HV); *Lotus japonicus* (Regel) K. Larsen (LJ); *Nicotiana tabacum* L. (NT); *Phytolacca americana* L. (PA); *Phytolacca dodecandra* L’Hér. (PD); *Pisum sativum* L. (PS); *Ricinus communis* L. (RC); *Triticum aestivum* L. (TA); *Vicia lens* (L.) Coss. and Germ. (VL); *Vicia sativa* L. (VA); *Zea mays* L. (ZM).

## 3. Mechanism of Ribosome Inactivation by RIPs

The mechanism of ribosome inactivation by RIPs has been known since 1987 [51,52]. RIPs are 28S rRNA *N*-glycosylases (EC 3.2.2.22) that catalyze the hydrolysis of the *N*-glycosidic bond of adenosine 4324 in the sarcin-ricin loop (SRL) of the large RNA of the 60S subunit of rat ribosomes or the equivalent in sensitive ribosomes of other organisms [30]. The SRL is part of the GTPase-associated center (GAC), which is the landing platform for translational GTPases (trGTPases) such as the prokaryote elongation factors EF-Tu and EF-G, and their eukaryote counterparts eEF1A and eEF2 [53]. The GAC consists of the SRL and the ribosomal stalk. The ribosomal stalk consists of a base made up of two ribosomal proteins and the lateral elements that are made up of several copies of proteins. The SRL and the base of the ribosomal stalk are conserved in prokaryotes and eukaryotes, while the lateral proteins are not conserved and are precisely the docking points of trGTPases and RIPs [54,55]. This may be the basis for the different specificity of RIPs for ribosomes of different species. The removal of adenine from the SRL, which is essential for the binding of trGTPases and even appears to be involved in the catalysis process, irreversibly inactivates ribosomes and has been reported to prevent eEF2 binding and GTP hydrolysis in eukaryotes [54].

A similar effect is caused by ribotoxins, such as α-sarcin, which are a group of extracellular ribonucleases that show cytotoxic activity towards animal cells [56,57]. These proteins are highly specific rRNA endonucleases (EC 4.6.1.23) that catalyze the hydrolysis of the phosphodiester bond between guanosine 4325 and adenosine 4326 in the SRL of rat 28S rRNA [58] (or the equivalent phosphodiester bond in ribosomes of other organisms), which prevents the binding of elongation factors [59]. Ribotoxins are produced by a few species of ascomycetes, mostly from the genus *Aspergillus* [57]. Due to the translation inhibitory and apoptotic activities of ribotoxins, they have been used as components of immunotoxins [57].

Finally, it should be noted that some RIPs can also remove more than one adenine from rRNA and many of them can catalyze the deadenylation not only of rRNA but also of other polynucleotide substrates such as DNA, poly(A), mRNA, tRNA, and viral RNA, and because of this, the name adenine polynucleotide glycosylase (or polynucleotide: adenosine glycosidase) was proposed for RIPs [12,60]. In addition, other activities have been reported for RIPs that could play a role in their possible function as defense elements [12,17].

## 4. RIP-like Proteins and Ribotoxin-like Proteins

There are plant proteins that have rRNA *N*-glycosylase activity but do not show homology or structural similarity to type 1 RIPs [61], and have been classified under various names (e.g., “small RIPs”, “small RIP 1 candidates”, or “RIP-Like Proteins”). These proteins could therefore exhibit all or at least some of the biological properties of RIPs and could also be used as crop defense tools. However, it should be noted that, although they inhibit protein synthesis, the *N*-glycosylase assay has not been performed on all of them, so some may have a different enzymatic activity. In fungi, some proteins with rRNA *N*-glycosylase activity have also been found without homology or structural similarity to plant RIPs [62,63], some of which have shown antifungal activity.

Ribotoxins have been found exclusively in ascomycetes; however, recently, proteins with the same activity have been found in basidiomycetes, and because they are not homologous with ascomycete ribotoxins they have been named Ribotoxin-Like Proteins (RLPs) [64]. As will be discussed later, some of them have shown antifungal activity.

## 5. Endocytosis of Ribosome-Inactivating Proteins

A very important question is how RIPs enter cells to carry out their enzymatic activity on ribosomes, since cytotoxic activity depends more on their ability to access ribosomes than on their catalytic power [16,28]. RIPs must interact with the cell membrane, and, following initial internalization, they are transported within the cell to the particular membrane where toxin translocation to the cytosol occurs. In addition, to enter fungal cells, RIPs must pass through the fungal cell wall. The internalization routes of RIPs have been studied in animal cells, where it has been observed that they can follow different internalization routes. In this respect, the most studied is ricin, a type 2 RIP that is highly toxic to animal cells. At picomolar concentrations, it binds to plasma membrane glycoproteins and is internalized into the cell [16,65]. Some protein molecules are recycled back to the plasma membrane, others undergo degradation in lysosomes, and a small number are transported first to the Golgi network and then to the endoplasmic reticulum. In the endoplasmic reticulum, the disulfide bridge is reduced, and the A-chain is translocated to the cytosol via the endoplasmic reticulum-associated degradation (ERAD) pathway. Finally, in the cytosol, the A-chain inactivates ribosomes, leading to cell death. Nigrin b and other type 2 RIPs from species of the genus *Sambucus*, which are thousands of times less toxic than ricin, can bind to plasma membrane glycoproteins other than ricin and internalize into the cell. All protein molecules are recycled back to the plasma membrane or transported to lysosomes for degradation. However, at a much higher extracellular concentration (40,000-fold), saturation of the endosome with nigrin b can lead to spontaneous release of nigrin b into the cytosol, causing inactivation of the ribosomes [16]. Type 1 RIPs such as saporin, trichosanthin, and curcins enter by binding to receptors of the LDLR (low-density lipoprotein receptor) family [66,67] and also follow different routes to those of ricin, most of them being located in endosomal compartments, which causes them to only reach the ribosomes at much higher concentrations than those required for ricin [66,68,69].

The internalization routes of RIPs have not been studied in fungi, but, given that they also possess endocytic mechanisms similar to those of animal cells [70,71], it would be expected that the routes of access to ribosomes would be similar to those discovered in animal cells. In addition, the cell wall represents an important barrier to the passage of macromolecules [72], which, given the diversity of the composition and structure of the cell walls of different fungi [73], could be an important element explaining, in part, the different sensitivities of fungi to different RIPs.

## 6. Inhibition of Fungal Growth by RIPs

At least 34 fungal species are sensitive to some RIPs or RIP-Like Proteins (Table 2). This includes a wide variety of species belonging to various families of basidiomycetes and ascomycetes. Furthermore, three species of fungus-like organisms are also sensitive to RIPs; thus, it has been reported that ME1 and ME2 from the roots of *Mirabilis expansa* (Ruiz and Pav.) Standl. slightly inhibit the growth of *Globisporangium irregulare* (Buisman) Uzuhashi, Tojo and Kakish, and *Phytophthora drechsleri* Tucker [27], and that the RIP isolated from the sarcocarp of *Cucurbita moschata* Duchesne strongly inhibits the growth of *Phytophthora infestans* (Mont.) de Bary [74]. Therefore, growth inhibition by the different RIPs could cover a spectrum that practically encompasses the entire fungal kingdom. The fungi on which RIPs have been tested are plant pathogens, although some of them, such as those of the genus *Aspergillus*, may be opportunistic pathogens in humans [75].

The antifungal activity of at least 20 RIPs obtained from 17 different species has been demonstrated (Table 3 and Table 4). This includes type 1 RIPs from two Poaceae (barley and maize) and several dicots and a type 2 RIP from the dicot *Sambucus nigra* L.

The antifungal activity of RIPs has been demonstrated both in vitro assays (Table 3) and, as discussed later, in transgenic plants (Table 4). Different types of in vitro assays have been used, thus, the RIP 30 from barley has been tested on microtiter plates and by different types of assay on agar plates. Sensitivity seems to be higher on microtiter plates than on agar plates [76]. Thus, inhibition of *Trichoderma reesei* E. G. Simmons growth was seen with a concentration of 11 μg/mL of RIP 30 on microtitre plates, whereas discs impregnated with 15 times more concentration are needed to obtain the same result [77]. In any case, RIP 30 was shown to inhibit the growth of several fungi: *Rhizoctonia solani* J.G. Kühn [76], *T. reesei* [76,77], *Fusarium sporotrichioides* Sherb. [76], and *Botrytis cinerea* Pers. [76]. However, about 16 fungal species have shown resistance to this RIP when tested on agar plates, including *Phycomyces blakesleeanus* Burgeff, *Alternaria alternariae* (Cooke) Woudenb. and Crous, and *Neurospora crassa* Shear and B.O. Dodge [77].

**Table 2:** Fungi that have been described as sensitive to RIPs or RIP-Like Proteins.

Order	Family	Species	References
Division Basidiomycota			
CLASS AGARICOMYCETES			
Cantharellales	Ceratobasidiaceae	*Rhizoctonia solani* J.G. Kühn	[28,76,78,79,80,81,82,83,84,85,86,87]
Polyporales	Polyporaceae	*Ganoderma boninense* Pat.	[88]
Agaricales	Agaricaceae	*Coprinus comatus* (O.F. Müll.) Pers.	[89,90]
Division Ascomycota			
CLASS LEOTIOMYCETES			
Helotiales	Erysiphaceae	*Blumeria graminis* (DC.) Speer	[91]
	Sclerotiniaceae	*Botrytis cinerea* Pers.	[76,87,92]
		*Clarireedia homoeocarpa* (F.T. Benn.) L.A. Beirn, B.B. Clarke, C. Salgado and J.A. Crouch	[93]
		*Sclerotinia sclerotiorum* (Lib.) de Bary	[94]
CLASS SORDARIOMYCETES			
Amphisphaeriales	Pestalotiopsidaceae	*Pestalotia* sp.	[95]
Diaporthales	Cryphonectriaceae	*Cryphonectria parasitica* (Murrill) M.E. Barr	[96]
	Valsaceae	*Cytospora* sp. *	[95]
Glomerellales	Plectosphaerellaceae	*Verticillium dahliae* Kleb.	[27]
Hypocreales	Hypocreaceae	*Trichoderma reesei* E.G. Simmons	[27,95]
		*Trichoderma harzianum* Rifai	[27]
	Nectriaceae	*Fusarium culmorum* (Wm.G. Sm.) Sacc.	[97]
		*Fusarium fujikuroi* Nirenberg	[98,99]
		*Fusarium graminearum* Schwabe	[94]
		*Fusarium oxysporum* Schltdl.	[27,90,95,100,101,102]
		*F. proliferatum* (Matsush.) Nirenberg ex Gerlach and Nirenberg	[27]
		*Fusarium sporotrichioides* Sherb.	[76]
Magnaporthales	Pyriculariaceae	*Pyricularia grisea* Cooke ex Sacc.	[103]
		*Pyricularia oryzae* Cavara	[104,105]
Sordariales	Sordariaceae	*Neurospora crassa* Shear and B.O. Dodge **	[77]
Xylariales	Hyponectriaceae	*Physalospora pyricola* Nose	[89,90]
CLASS EUROTIOMYCETES			
Eurotiales	Aspergillaceae	*Aspergillus flavus* Link	[94,106]
		*Aspergillus nidulans* (Eidam) G. Winter	[106]
		*Aspergillus niger* Tiegh.	[94,107]
		*Penicillium digitatum* (Pers.) Sacc.	[29,30,108,109]
CLASS DOTHIDEOMYCETES			
Pleosporales	Corynesporascaceae	*Corynespora cassiicola* (Berk. and M.A. Curtis) C.T. Wei	[110]
	Didymellaceae	*Didymella arachidicola* (Khokhr.) Tomilin	[90,101]
		*Phoma* sp.	[95]
	Pleosporaceae	*Alternaria alternata* (Fr.) Keissl.	[92]
		*Alternaria brassicae* (Berk.) Sacc.	[111]
		*Alternaria solani* Sorauer	[27,102]
		*Cochliobolus heterostrophus* (Drechsler) Drechsler	[94]

* Sensitive only to RIP-Like Proteins; ** Mutant os-1. Synonyms used in the cited articles: *Blumeria graminis* (DC.) Speer (=*Erysiphe graminis* DC.); *Fusarium fujikuroi* Nirenberg (=*Fusarium verticillioides* (Sacc.) Nirenberg); *Clarireedia homoeocarpa* (F.T. Benn.) L.A. Beirn, B.B. Clarke, C. Salgado and J.A. Crouch (=*Sclerotinia homoeocarpa* F.T. Benn.); *Pyricularia grisea* Cooke ex Sacc. (=*Magnaporthe grisea* (T.T. Hebert) M.E. Barr); *Cytospora* sp. (Cytospora canker); *Globisporangium irregulare* (Buisman) Uzuhashi, Tojo and Kakish. (=*Pythium irregulare* Buisman); *Fusarium oxysporum* Schltdl. (=*Fusarium oxysporum* var. solani Raillo); *Cochliobolus heterostrophus* (Drechsler) Drechsler (=*Bipolaris maydis* (Y. Nisik. and C. Miyake) Shoemaker); *Aspergillus flavus* Link (=*Aspergillus oryzae* (Ahlb.) Cohn); *Cucumis melo* L. (=*Luffa cylindrica* M.Roem.); *Didymella arachidicola* (Khokhr.) Tomilin (=*Mycosphaerella arachidicola* Khokhr.).

**Table 3:** Ribosome-inactivating proteins that inhibit fungal growth in vitro. The RIPs, the families and species from which they have been obtained, and the fungi in which this activity has been demonstrated are shown.

Species and RIP	Fungi	Ref.
POACEAE		
*Hordeum vulgare* L.		
Barley RIP30	*Botrytis cinerea*, *Fusarium sporotrichioides*, *Neurospora crassa* *, *Rhizoctonia solani*, *Trichoderma reesei*	[76,77]
*Zea mays* L.		
Maize b-32 (MOD1)	*Aspergillus flavus*, *A. nidulans*, *R. solani*	[80,106]
AMARANTHACEAE		
*Salsola soda* L.		
Sodin 5	*Penicillium digitatum*	[109]
*Chenopodium quinoa* Willd.		
Quinoin	*Cryphonectria parasitica*, *P. digitatum*	[96,109]
*Beta vulgaris* L.		
BE27	*P. digitatum*	[29,108]
PHYTOLACCACEAE		
*Phytolacca dioica* L.		
Dioicin 2	*P. digitatum*	[30]
PD-S2	*P. digitatum*	[30]
*Phytolacca heterotepala* H.Walter		
PhRIP I	*B. cinerea*	[92]
NYCTAGINACEAE		
*Mirabilis expansa* (Ruiz and Pav.) Standl.		
ME1 and ME2	*Alternaria solani*, *Fusarium oxysporum*, *F. proliferatum*, *Globisporangium irregulare*, *Phytophthora drechsleri*, *R. solani, Trichoderma harzianum*, *T. reesei*, *Verticillium dahliae*	[27,28]
CUCURBITACEAE		
*Momordica charantia* L.		
Alpha-momorcharin (α-MMC)	*A. flavus*, *A. niger, Cochliobolus heterostrophus*, *Fusarium graminearum*, *F. oxysporum*, *F. solani, Pyricularia oryzae*, *Sclerotinia sclerotiorum*	[94,100,105]
*Momordica balsamina* L.		
MbRIP-1	*Aspergillus niger*	[107]
*Benincasa hispida* Cogn.		
Hispin	*Coprinus comatus*, *Didymella arachidicola*, *F. oxysporum*, *Physalospora pyricola*	[90]
SOLANACEAE		
*Nicotiana tabacum* L.		
TRIP	*C. heterostrophus*, *Cytospora* sp., *F. oxysporum*, *Pestalotia* sp., *Phoma* sp., *T. reesei*	[95]
ARECACEAE		
*Elaeis guineensis* Jacq.		
EgRIP-1a and EgRIP-1b	*Ganoderma boninense*	[88]
VIBURNACEAE		
*Sambucus ebulus* L.		
Pebulin	*A. solani*, *F. oxysporum*	[102]

* Mutant os-1.

Notably, the protoplast-forming mutant os-1 of *N. crassa* was sensitive to RIP 30, indicating that, at least in this organism, the presence of an intact cell wall protects against the antifungal activity of the RIP [77]. *N. crassa* ribosomes were approximately 10 times more sensitive to inactivation than ribosomes from ascites cells [77], indicating that it is not the sensitivity of the ribosomes to RIP that determines their toxicity but rather their ability to reach the ribosomes. The inhibition of *T. reesei* growth by RIP 30 was enhanced in the presence of both chitinase and barley β-1,3-glucanase, whereas with *F. sporotrichioides* it was only enhanced in the presence of chitinase [76]. This synergistic inhibition suggests that inhibition by RIP 30 is enhanced when hyphal cell walls are permeabilized by the action of these hydrolases.

**Table 4:** Transgenic fungus-resistant plants bearing RIP genes.

RIP	Host	Pathogen	Ref.
Barley RIP30	*Nicotiana tabacum* L.	*Rhizoctonia solani*	[78,79]
	*Triticum aestivum* L.	*Blumeria graminis*	[91]
	*Solanum tuberosum* L.	*R. solani*	[86,112]
	*Brassica juncea* (L.) Czern.	*Alternaria brassicae*	[111]
	*Vigna mungo* (L.) Hepper	*Corynespora cassiicola*	[110]
Maize b-32	*N. tabacum*	*R. solani*	[80]
	*T. aestivum* L	*Fusarium culmorum*	[97]
	*Zea mays* L.	*Fusarium fujikuroi*	[98]
MOD1	*Oryza sativa* L.	*R. solani*	[84]
	*Z. mays*	*F. fujikuroi*	[99]
PAP (PAP I)	*N. tabacum*	*R. solani*	[81,83]
PAPII	*N. tabacum*	*R. solani*	[82]
	*Agrostis stolonifera* L.	*Clarireedia homoeocarpa*	[93]
PhRIP I	*N. tabacum*	*Botrytis cinerea*, *Alternaria alternata*	[92]
	*S. tuberosum*	*B. cinerea*, *R. solani*	[87]
TCS	*O. sativa*	*Pyricularia oryzae*	[104]
α-MMC	*O. sativa*	*Pyricularia grisea*	[103]
Curcin 2	*N. tabacum*	*R. solani*	[85]

*Aspergillus flavus* Link (which is not an aggressive pathogen of maize, but has a great economic impact due to its production of aflatoxin) and *Aspergillus nidulans* (Eidam) G. Winter were sensitive to MOD1 (RIP1), i.e., an engineered form of maize RIP b-32 (proRIP1) that does not require proteolytic activation [106]. MOD1 not only affected the growth of the fungi but also altered their morphology. The growth inhibition was concentration-dependent, being evident at 200 μg/mL, and above. *R. solani* was more sensitive to RIP b-32; from 0.6 µg/mL, the growth inhibition was shown in a microtiter plate assay [80].

In dicotyledons, type 1 RIPs with antifungal activity have been found in species of the families Amaranthaceae, Phytolaccaceae, Nyctaginaceae, Cucurbitaceae, Solanaceae, and Arecaceae (Table 3).

*Penicillium digitatum* (Pers.) Sacc. was very sensitive to different type 1 RIPs: sodin 5 [109], quinoin [109], BE27 [29,108], dioicin 2 [30], and PD-S2 [30]. The one that exerted the greatest effect was BE27 since the growth inhibition was evident at 0.6 µg/mL [29]. The other RIPs mentioned inhibited fungal growth from concentrations of 5–10 μg/mL; however, PD-L4 did not inhibit fungal growth at 30 μg/mL [30]. Notably, like sodin 5, quinoin, BE27, diocin 2, and PD-S2, PD-L4 also have *N*-glycosylase activity on yeast ribosomes [30,109]. BE27, diocin 2, PD-S2, and PD-L4 have also been reported to be active against *P. digitatum* ribosomes [30].

The fact that PD-L4 does not show antifungal activity [30] despite showing high homology with PD-S2 [113] suggests that entry into cells may be the limiting step for the fungicidal capacity of RIPs, and it has been suggested that the amphipathicity of the carboxyl-terminal domain could play a relevant role in the different degrees of toxicity of RIPs towards fungi [30]. In fact, BE27, which is the most toxic to *P. digitatum*, is the one with the highest degree of amphipathicity in the carboxyl-terminal domain and has been shown to be able to internalize and depurinate fungal ribosomes [29]. This is in agreement with the studies reported with RIPs obtained from *M. expansa*. ME1 and ME2 are two RIPs, obtained from the root of *M. expansa*, which showed both rRNA *N*-glycosylase activity on yeast ribosomes and antifungal activity in an agar plate assay against various fungi (Table 3), but were shown to be inactive against others [27]. Park et al. [28] compared the activity of three RIPs (ricin A-chain, saporin-S6, and ME from *M. expansa*) on fungal ribosomes and their antifungal activity. Ricin A-chain and saporin-S6 were much more active on ribosomes of *Alternaria solani* Sorauer, *R. solani*, *T. reesei*, and *Candida albicans* (C.P. Robin) Berkhout than the RIP of *M. expansa*; however, this was the only one able to inhibit the growth of *R. solani* because it was the only one able to enter the fungus.

Quinoin also induced a slight inhibition of the growth of *Cryphonectria parasitica* (Murrill) M.E. Barr [96]. Another RIP from Phytolacaceae, PhRIP I, was able to inhibit the germination of *B. cinerea* spores [92].

Alpha-momorcharin (α-MMC), a RIP from *Momordica charantia* L. seeds, inhibited sporulation [94] or mycelial growth [100,105] of a wide variety of pathogenic fungi (Table 3); however, it was ineffective in inhibiting the growth of *C. albicans* [100], supporting the hypothesis that antifungal activity also depends on the fungus studied. Interestingly, α-MMC, in addition to antifungal activity, also has antibacterial [100] and antiviral [12] activity, making it an excellent tool for crop protection against a wide variety of pathogens. Two other RIPs obtained from cucurbits, MbRIP-1 [107] and hispin [90], have antifungal activity. The latter, despite inhibiting the growth of several fungi (Table 3), was shown to be ineffective against *B. cinerea* [90].

The other type 1 RIPs that have shown antifungal activity are TRIP from tobacco [95] and two isoforms, EgRIP-1a and EgRIP-1b, obtained from oil palm (*Elaeis guineensis* Jacq.) [88]. TRIP showed rRNA *N*-glycosylase activity against yeast and *T. resei* ribosomes and presented growth inhibitory activity against *T. resei* and other fungi in agar plate assays [95]. The activity was different in different fungal species and, in some cases, was ineffective. Partially purified oil palm RIPs showed rRNA *N*-glycosylase activity on yeast ribosomes and inhibited the growth of *Ganoderma boninense* Pat., an oil palm pathogen causing basal stem rot (BSR) [88]. The only type 2 RIP reported to have antifungal activity is pebulin, a recombinant protein from *Sambucus ebulus* L. This protein was able to completely inhibit the germination of *A. solani* and *Fusarium oxysporum* Schltdl. spores at a concentration of 5 µg/mL [102].

In addition, peptides of around 10 kDa obtained from the cucurbits *Cucumis melo* L. and *Benincasa hispida* Cogn. have been reported as RIPs with antifungal activity [89,101]. On the other hand, the ribotoxin α-sarcin [108] and the RLPs ageritin [114,115] and eryngitins 3 and 4 [116] have also shown antifungal activity, indicating that, although never used for that purpose, these proteins could also be tools to defend crops against fungal diseases.

## 7. Mechanisms of Antifungal Activity

**Figure 1:**
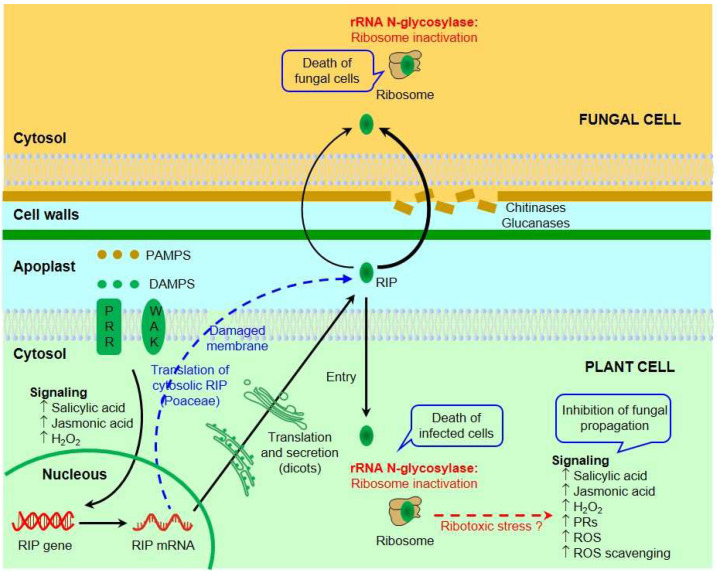
Proposed mechanisms for the antifungal activity of RIPs. The infection causes the release of PAMPs that are recognized by PRRs and DAMPs which, in turn, are recognized by WAKs, leading to an increase in signal molecules, inducing RIP expression. Type 1 RIPs from dicots are synthesized in the endoplasmic reticulum and are localized in the apoplast. Infection by pathogens can alter the permeability of the host cell membrane, allowing RIP to enter the cytoplasm and inactivate ribosomes, leading to cell death, which prevents the spread of the pathogen. RIP can also pass through the cell wall and membrane of the fungus, inactivating its ribosomes and causing its death. In the case of cytosolic RIPs (Poaceae), these may be released as a consequence of fungal damage to the cell membrane. Chitinases and glucanases can degrade the fungal cell wall and favor RIP entry. RIP can also trigger fungal defense signaling pathways. The activation of these pathways could be a consequence of ribotoxic stress caused by RIPs.

Several mechanisms have been proposed for the antifungal action of RIPs (Figure 1).

Since many RIPs inactivate plant ribosomes (Table 1), it has been proposed that they could be part of a “suicide mechanism” [32,33,117]. The RIPs that have been reported to have antifungal activity are, except pebulin, type 1 RIPs (Table 3 and Table 4). RIPs from Poaceae do not have a leader peptide [118] and are localized in the cytoplasm [47,49]. However, it seems that these RIPs do not affect the ribosomes of the same plant (Table 1). Some are synthesized as precursors and subsequently undergo processing, but this does not appear to significantly increase enzymatic activity against their own plant ribosomes [49]. Many type 1 RIPs from dicots are potent inhibitors of protein synthesis in plants (Table 1), but, having leader peptides [118], they are synthesized in the endoplasmic reticulum, and are located in the apoplast, the space between the plasma membrane and the cell wall [34,117,119], thus avoiding contact with ribosomes. Therefore, it has been assumed that pathogen infection would alter the permeability of the host cell membrane, allowing RIPs access to ribosomes and leading to the arrest of protein synthesis and cell death. This would prevent the spread of the pathogen throughout the rest of the plant [32,33,117]. In addition, RIP expression could be increased by the presence of the pathogen, since infection causes the release of pathogen-associated molecular patterns (PAMPs) that are recognized by pattern recognition receptors (PRRs) and damage-associated molecular patterns (DAMPs) that are recognized by wall-associated kinases (WAKs) leading to the increase of defense signal molecules such as hydrogen peroxide, salicylic acid, or jasmonic acid [120]. Hydrogen peroxide and salicylic acid have been shown to increase BE27 expression [29,34], jasmonic acid has been shown to increase the expression of α-momorcharin (α-MMC) [121], and methyl jasmonate and salicylic acid have been shown to increase curcin-L expression [122]. These are type 1 RIPs that are expressed in *Beta vulgaris* leaves, in different tissues of *M. charantia*, and in *Jatropha curcas* L. leaves, respectively.

A second mechanism Involves a direct effect on pathogen ribosomes. Many, but not all, RIPs inhibit the growth of various fungi in vitro (Table 3). Such inhibition appears to be related to the ability of the RIP to reach ribosomes by traversing the fungal cell wall and membrane [28,29]. Chitinases and glucanases can degrade the fungal cell wall and favor RIP entry, as these enzymes have been shown to enhance the antifungal capacity of some RIPs [76].

The third proposed mechanism involves the generation of signaling molecules that defend the plant from attack by fungi and other pathogens [120,123]. Not all RIPs generate the same signals and different results have been obtained depending on the RIP and the plant studied. Thus, it has been reported that α-MMC, in *Nicotiana benthamiana* Domin plants sprayed with a solution of the RIP, up-regulates the expression of genes related to the scavenging of reactive oxygen species (ROS), modulating ROS homeostasis, and some defense-related genes responsive to salicylic acid [94,124], and that in *Nicotiana tabacum* L. plants it induces an increase in both jasmonic acid and salicylic acid [125]. In contrast, the same RIP sprayed on *M. charantia* plants increases jasmonic acid biosynthesis and ROS induction without a relevant increase in salicylic acid [121]. PAP and PAPII (two type 1 RIPs, obtained from spring and early summer leaves of *P. amaricana*, respectively) generate a signal leading to overexpression of pathogenesis-related proteins in the absence of increased salicylic acid levels, making transgenic tobacco plants resistant to virus and fungal infection [81,82,83,126].

The relationship between the enzymatic activity of RIPs and their ability to induce the production of signaling molecules in plants has not been studied. In animals, ricin, α-sarcin, and Shiga toxin, as a consequence of their enzymatic action on the sarcin-ricin loop (SRL), activate signaling pathways through the mitogen-activated protein kinases (MAPKs) p38 and JNK [127]. Deoxynivalenol (DON) and T-2 toxin (both trichothecene mycotoxins) inhibit protein synthesis and induce ERK1/2 and p38 MAPK activation in several cell lines, followed by increased cytokine production [128]. This ribosome-mediated MAPK activation is termed “ribotoxic stress response” [128]. In *Arabidopsis thaliana* (L.) Heynh., DON, and T-2 toxin induce the expression of MPK3 and MPK6, which are implicated as positive regulators of the hypersensitive response through ethylene and ROS signaling [128]. It is therefore possible that the generation of signaling compounds by plants is a response to the ribotoxic stress produced by RIPs.

In conclusion, RIPs could exert their antifungal action through various mechanisms. Probably, depending on the RIP and the pathogen, one mechanism could predominate over the others or the effect could be a combination of several of them.

## 8. Transgenic Plants Resistant to Fungal Infection

Genetic engineering has proved to be an excellent method for obtaining fungus-resistant plants [120,129,130]. In this way, plants expressing genes that protect the plant from fungal infections have been obtained. Using this strategy, transgenic plants have been designed that carry the gene for a RIP and are resistant to pathogenic fungi that cause disease (Table 4). The most commonly used model is the tobacco plant (*N. tabacum*), but plants have also been obtained from some important crops such as wheat (*Triticum aestivum* L.), potato (*Solanum tuberosum* L.), Indian mustard (*Brassica juncea* (L.) Czern.), black gram (*Vigna mungo* (L.) Hepper), maize (*Zea mays* L.), rice (*Oryza sativa* L.), or creeping bentgrass (*Agrostis stolonifera* L.), widely used as turf (Table 4).

**Figure 2:**
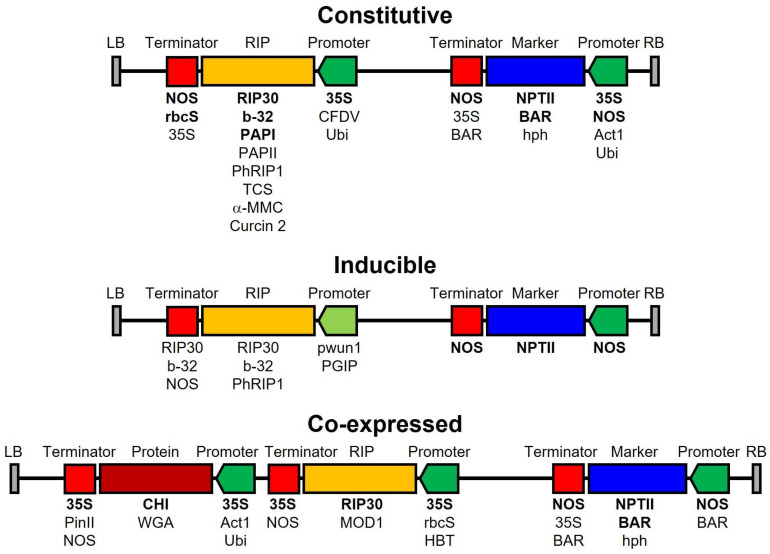
Strategies used for the construction of the T-DNA region of binary vectors for plant transformation with *A. tumefaciens*. Vectors with constitutive promoters, inducible promoters, and vectors expressing RIPs and chitinases or wheat germ agglutinin (WGA) have been designed. LB: left border, RB: right border, NOS: nopaline synthase, rbcS: rice rbcS gene, 35S: cauliflower mosaic virus (CMV), CFDV: coconut foliar decay virus, Ubi: ubiquitin, BAR: bar gene (resistance to glufosinate), NPT II: neomycin phosphotransferase II (resistance to kanamycin and neomycin), hph: hygromycin phosphotransferase gene (hygromycin resistance), Act1: rice actin 1, pwun1: promoter of the potato wun1 gene (wound-inducible), PGIP: bean polygalacturonase gene I promoter, PinII: 3’ region of the potato proteinase inhibitor II gene, CHI: chitinase, WGA: wheat germ agglutinin, HBT: HBT promoter (of the C4PPDK gene). The most used elements are written in bold.

The most commonly used method to obtain these transgenic plants is *Agrobacterium tumefaciens*-mediated transformation, although direct methods such as biolistics [91,93,97,104] and polyethylene glycol-mediated transfer [98] have also been used. Different RIPs have been constitutively expressed under the control of a strong promoter such as the cauliflower mosaic virus promoter (CaMV 35S) (Figure 2), since there is a strong correlation between the level of RIP expression and the level of resistance against the fungi [85]. Genes conferring resistance to kanamycin and neomycin, hygromycin, or glufosinate (Figure 2), also controlled by strong promoters, are used as selection marker genes.

Although cases have been reported in which the transgenic plants show a normal phenotype [85,87,91,104], or at most a slightly smaller size [97], in other cases the constitutively expressed RIPs were toxic to the plants, probably due to their ability to inactivate host plant ribosomes [81,83,93]. This major drawback has been overcome by using the RIP gene with the sequence that directs it to the apoplast [85], by introducing mutations that reduce RIP toxicity without affecting its antifungal activity [81,83,93], or by using RIP genes that are less toxic to plants [82]. The latter may depend on the RIP and the host, e.g., PAPII has been reported to be toxic to creeping bentgrass [93], but not to tobacco [82]. This is also true for the RIP-pathogen relationship, so it has been reported that transgenic plants resistant to one fungus are not resistant to others [84,112]. Another strategy is to use inducible promoters that respond to the damage caused by the fungus in the plant (Figure 2) so that RIP is only expressed when the plant is attacked by the fungus without affecting its development [78,80,87,92].

To enhance the antifungal activity of RIPs, genes encoding chitinases have also been introduced [79,84,86,99,110,111], which as we have seen exert a synergistic effect with RIPs, and even the lectin WGA [99] (Figure 2). In addition, some transgenic plants carrying RIPs are also resistant to viruses and insects [9,12], which adds even more interest to this type of strategy. For example, tobacco plants carrying the PAP II gene are resistant to the fungus *R. solani* and the viruses TMV and PVX [82]; those carrying curcin 2 are resistant to *R. solani* and TMV [85]; and maize plants carrying maize ribosome-inactivating protein (MRIP), tobacco hornworm chitinase (THWC), and wheat germ agglutinin (WGA) are resistant to the fungus *Fusarium fujikuroi* Nirenberg and the insects *Spodoptera frugiperda* Walker and *Helicoverpa zea* Boddie [99].

## 9. Conclusions

The use of ribosome-inactivating proteins (RIPs) is a promising alternative to chemical-based fungicides, which cause problems such as environmental contamination, resistant development, and residual toxicity. The fact that RIPs also possess antiviral and insecticidal activities makes them an ideal tool for disease and pest control in crops. 

In view of the published results, it seems that the most efficient way to use these proteins would be to construct transgenic plants carrying genes for RIPs and genes for other defense proteins with which they show a synergistic effect.

However, in order to use these proteins effectively, further studies are still needed to shed light on the toxicity of the different RIPs to the host plants, the efficacy of each RIP on the fungi causing the diseases to be controlled, the synergistic effect with other fungicidal agents, as well as the mechanisms of antifungal action.

## Data Availability

Not applicable.
